# Translational activity is uncoupled from nucleic acid content in bacterial cells of the human gut microbiota

**DOI:** 10.1080/19490976.2021.1903289

**Published:** 2021-03-28

**Authors:** Mariia Taguer, B. Jesse Shapiro, Corinne F. Maurice

**Affiliations:** a Department of Microbiology & Immunology, Faculty of Medicine, McGill University, Montreal, Quebec, Canada; b Department of Microbiology & Immunology, McGill Genome Centre, Faculty of Medicine, McGill University, Montreal, Quebec, Canada

**Keywords:** Gut microbiome, flow cytometry, bacterial physiology, bacterial activity, BONCAT, single-cell

## Abstract

Changes in bacterial diversity in the human gut have been associated with many conditions, despite not always reflecting changes in bacterial activity. Methods linking bacterial identity to function are needed for improved understanding of how bacterial communities adapt and respond to their environment, including the gut. Here, we optimized bioorthogonal non-canonical amino acid tagging (BONCAT) for the gut microbiota and combined it with fluorescently activated cell sorting and sequencing (FACS-Seq) to identify the translationally active members of the community. We then used this novel technique to compare with other bulk community measurements of activity and viability: relative nucleic acid content and membrane damage. The translationally active bacteria represent about half of the gut microbiota, and are not distinct from the whole community. The high nucleic acid content bacteria also represent half of the gut microbiota, but are distinct from the whole community and correlate with the damaged subset. Perturbing the community with xenobiotics previously shown to alter bacterial activity but not diversity resulted in stronger changes in the distinct physiological fractions than in the whole community. BONCAT is a suitable method to probe the translationally active members of the gut microbiota, and combined with FACS-Seq, allows for their identification. The high nucleic acid content bacteria are not necessarily the protein-producing bacteria in the community; thus, further work is needed to understand the relationship between nucleic acid content and bacterial metabolism in the human gut. Considering physiologically distinct subsets of the gut microbiota may be more informative than whole-community profiling.

## Introduction

The human gut microbiota is comprised of trillions of microorganisms that together help maintain gut homeostasis and provide key ecosystem services to the human host such as nutrient digestion, pathogen exclusion, and immune system development.^1,2^ The diversity of the gut microbiome across different human populations and across various diseases has been well cataloged.^3–8^ Still, it is becoming increasingly clear that bacterial diversity and activity are not always coupled, and thus which members are key for maintaining host health cannot be elucidated through diversity metrics alone.^9–11^ New methods that link together bacterial function to bacterial identity are needed to further explore the role of the gut microbiome in health and disease.

The active subset of the gut microbiota is more sensitive and responsive to perturbations than the diversity of the whole community alone.^12,13^ Broad range ‘omics techniques such as metatranscriptomics, metabolomics, and metaproteomics provide an overall depiction of the gut microbiota’s output, yet incomplete functional databases make it challenging to link together bacterial identity to function.^14^ To characterize the active fraction of the gut microbiota, a broad and measurable indicator of activity needs to be identified that labels the active bacteria with minimal bias. Single-cell techniques allow for increased resolution of the heterogeneity of activity in complex microbial systems, helping determine the actual contribution of specific bacterial members *in situ*. Heavy water incorporation, substrate uptake, and nucleic acid content have all been studied in the context of the gut microbiota.^10,12,15–18^ Yet they have low throughput, are costly, or, for the case of nucleic acid content, the relevance to activity is unclear. Single-cell techniques to study bacterial physiology and activity have been well detailed by Hatzenpichler *et al* (2020).^19^

The use of relative nucleic acid content as a marker of bacterial activity was first introduced in aquatic systems, where the microbial community, when stained with a nucleic acid dye, clusters into two distinct populations based on their level of nucleic acid content.^20^ This was seen with a variety of nucleic acid dyes, such as SYBR Green I used in this study, which stains both DNA and RNA.^21^ The more fluorescent population consists of bacteria with higher nucleic acid content (HNA) than their low nucleic acid (LNA) counterparts. This phenomenon has since been widely studied, and is proposed to link together bacterial nucleic acid content to a gross level of bacterial metabolism, where the HNA bacteria are more metabolically active than the LNA. This has been demonstrated through higher leucine incorporation rates, ATP cell^−1^ concentrations, respiration rates, and proportions correlating with overall bacterial production,^22–28^ but these dynamics have been disputed as well.^29^

As this bimodal distribution of HNA and LNA has already been identified in the gut,^10^ we set out to determine if HNA and LNA components of the microbiota differ in their metabolic activity. To do so, a broad yet clearly defined measurement of single-cell levels of activity was required to compare and contrast with relative nucleic acid content determination. In this paper, we focus on protein translation as an important aspect of metabolic activity. We optimize bioorthogonal non-canonical amino acid tagging (BONCAT), a recent application of click chemistry, to identify the translationally active bacteria in the gut microbiota.^30^ BONCAT allows for the unbiased detection of proteins produced *in situ* under biologically relevant conditions, without the need for radioactivity, isotopes, antibodies, long incubations, or altering conditions. A methionine analogue, L-homopropargylglycine (HPG), is added to a short *in vitro* incubation of the gut microbiota, and is then “clicked” to an azide-modified fluorophore. HPG has been shown to be taken up by all bacteria under all physiological states tested, and due to the promiscuity of methionyl-tRNA synthetase, is incorporated into nascent proteins.^30^ The alkyne-azide groups quickly undergo a cycloaddition to form a stable triazole conjugate at biologically relevant conditions. Azide and alkyne modifications are considered biologically inert: they do not interfere with biological processes and do not naturally exist in most biological systems, including bacteria.^30–32^

Previously, BONCAT has been used in bacterial isolates, natural assemblages in aquatic systems,^31–33^ soil,^34^ and sputum from cystic fibrosis patients,^35^ and typically combined with fluorescent in-situ hybridization (FISH) with 16S rRNA probes to identify the protein-producing bacteria. To our knowledge, ours is the first study to apply BONCAT to the gut microbiota, while other gut microbiota studies have focused on selective uptake and incorporation.^36^ As 16S rRNA-FISH offers limited taxonomic resolution and requires designing probes for specific taxa of interest *a priori*,^37^ we instead combine BONCAT with fluorescence-activated cell sorting (FACS) and subsequent 16S rRNA gene sequencing (FACS-Seq) as previously done elsewhere.^34,35,38^ This allows us to identify the diversity of the BONCAT+ and BONCAT- communities, linking together bacterial identity to activity and increasing throughput. Assessing protein production through BONCAT yields similar results to assessing protein production through nano-SIMS^30^and MAR-FISH,^31^ yet it is faster and less expensive.

Applying BONCAT to the gut microbiota provides some unique challenges that we address in this study. The incorporation of HPG into nascent proteins requires a short *in vitro* incubation, which is difficult for the gut microbiota, as there is no single media that is able to support the growth of all gut bacteria. Indeed, “culturomics” is an ongoing, developing field to broaden our ability to culture more of the fastidious members of the gut microbiota.^39^ Furthermore, methionine is common in the gut, but inhibits HPG incorporation. HPG has an activation rate 500 times lower than methionine,^40^ and bacteria preferentially incorporate methionine over HPG ten-fold, so excess HPG is required to outcompete methionine.^31^ Methionine is expected to be ubiquitous in the gut lumen, and as such, fecal bacteria are not immediately well-suited for BONCAT. The approach detailed in this study addresses and overcomes some of these limitations.

Lastly, to explore what the inactive subset of the gut microbiota may represent, we identified the damaged subset of the gut microbiota with propidium iodide (PI), a membrane exclusion dye. By contrasting the active subset to the damaged subset, we hope to begin to explore if the less active or inactive fraction represents dormant bacteria acting as a seedbank, or external transient bacteria that are unable to colonize the gut.^41–45^

Here we show that the HNA and BONCAT+ bacteria are taxonomically distinct, suggesting that the HNA bacteria are not necessarily undergoing translation. We explore how the HNA bacteria contain more of the conserved bacteria across individuals, contributing to the “core” gut microbiome, while the BONCAT+ community is a subset of the whole community and potentially more responsive to changes in the environment.

## Results

### Optimizing incubation conditions for HPG labeling of gut bacteria

The BONCAT method requires an incubation step as bacteria incorporate HPG. In the interest of maintaining gut bacterial activity to levels as similar to *in situ* as possible, optimization of the media, length of incubation, and concentration of HPG was necessary to ensure that bacterial growth and community structure were not altered. To best mimic the gut environment, a fecal slurry was created by homogenizing fresh stool in reduced PBS (rPBS – 1 mg mL^−1^ L-cysteine), and the supernatant of the fecal slurry was tested as the media at varying concentrations, along with different incubation times. An additional challenge to an *in vitro* BONCAT incubation is that methionine is preferentially incorporated over HPG,^30,31^ and the gut is a nutritionally rich environment with high concentration of methionine (Table S1). As such, HPG was added in excess of 10X what is found in the gut to outcompete methionine incorporation.

**Figure 1.**
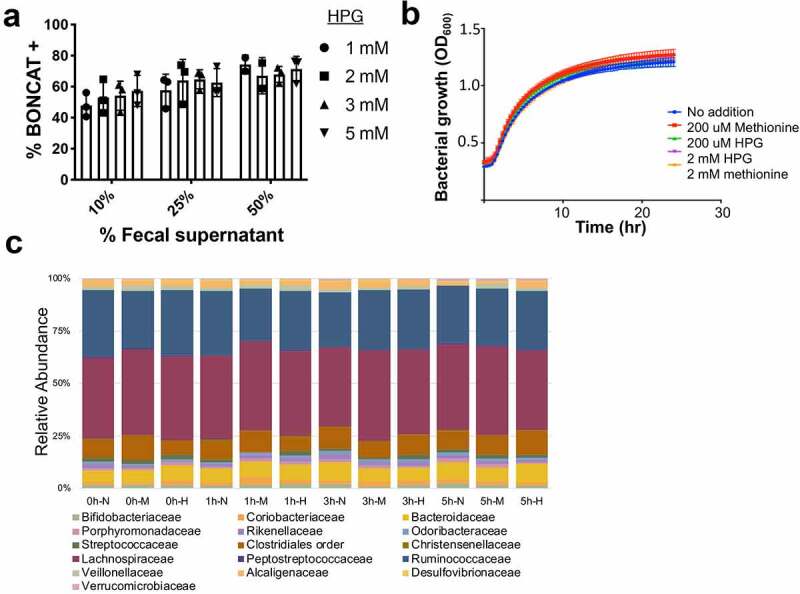
Optimizing*in-vitro* BONCAT incubation conditions for the human gut microbiota. a) HPG concentrations between 1 and 5 mM tested with varying concentrations of fecal supernatant between 10 and 50% show no significant differences in incubation based on HPG concentration. b) Growth curves with HPG, methionine, or neither. Bacteria were diluted 1/10 in 50% supernatant and incubated at anaerobically at 37°C in the dark, with shaking. c) Family level 16S rRNA gene sequencing of the gut microbiota with and without HPG or methionine in 50% supernatant for 1, 3, or 5 hours (n = 1). Labels indicate time (hours), followed by No addition (N), Methionine (M), or HPG (H)

Gut bacteria incubated with 2 mM HPG in 50% fecal supernatant for 2 hours results in saturated HPG incorporation without altering growth (Figure 1a,b) or community composition as per 16S rRNA gene sequencing (Figure 1c). The concentration of supernatant has a significant effect on the BONCAT signal (*p* = .032, 2-way ANOVA, Tukey’s multiple comparisons test), but HPG concentration does not, once above 1 mM (Figure 1a).

### Optimizing fluorescence-activated cell sorting (FACS) of BONCAT-labeled bacteria

To link bacterial identity to bacterial activity, we optimized a method to sort the translationally active bacteria (BONCAT+) and sequence with 16S rRNA gene amplification (BONCAT-FACS-Seq). We first verified the click protocol was able to capture all HPG-incorporating bacteria by using *Escherichia coli* in exponential phase as a positive control. Control incubations not containing the fluorophore (Alexa-azide 647) or HPG (Fig S1ab) were used to determine the appropriate gating for flow cytometric analysis of the BONCAT+ population, which was 96% BONCAT+ for *E. coli* in exponential phase with glucose supplementation (Fig S1c). For the gut microbiota, similar gating controls are employed in a consistent manner (Fig S1d) to determine the BONCAT+ population (Fig S1e). As shown elsewhere, dead bacteria did not uptake HPG^30,31^ (Fig S1f).

We determined that sorting 180,000 events was sufficient to represent the unsorted population. Based on a probability mass function for the binomial distribution, we modeled the probability of finding k bacteria from a total of N bacteria. Based off the cumulative probability of finding k or less from a sample size of N, we calculated the theoretical value that if we sort 25,000 events (N), we would capture 100 bacteria (k) that are present in the initial population at a prevalence of 0.5%. We then sorted a range of events from 50,000 to 1,000,000, confirming we sorted viable bacteria that resulted in amplifiable DNA (Fig S2ab). Sorted samples have a slightly lower alpha diversity than the unsorted samples (Fig S2c), yet still cluster near the unsorted samples based on Bray-Curtis dissimilarity in a principal coordinate analysis with no differences in community composition between sorted and unsorted (R^2^ = 0.027, *p* = .887, PERMANOVA)(Fig S2d). The sorting purity for BONCAT, as determined by re-acquiring the sorted fractions by flow cytometry, however, is lower than sorting with other physiological dyes such as SYBR Green I, with a mean purity of the BONCAT+ fraction at 80% ± 10% (Table S2). The BONCAT- fraction, however, has a higher purity level after sorting at 94.3% ± 9%.

The cell sorting process introduced contaminant DNA into the sorted samples. A negative control was sorted each sorting day, consisting of the cell sorter’s sheath fluid, and was extracted and sequenced alongside the samples. The sheath fluid-negative controls contained between 341 and 2,333 reads, mostly assigned to *Pseudomonas*. Thus, to remove sheath fluid contaminants from the samples, we removed reads that were present in the sheath fluid, but absent from the initial, unsorted sample from all samples. This had only a minor effect on the diversity and read count of most samples, the most pronounced effect being in the BONCAT- fraction (Fig S3). Thus, we conclude that BONCAT-FACS-Seq yields generally representative 16S communities of the BONCAT+ and BONCAT- populations and is an appropriate method moving forward.

### Diversity of physiologically distinct fractions of the gut microbiota

To determine the active and damaged members of the gut microbiota, we sorted bacteria based on the BONCAT signal, relative nucleic acid content (HNA and LNA), and membrane damage (PI+). Fresh fecal samples were obtained from ten healthy, unrelated individuals who had not received antibiotics in the past 3 months, and immediately placed in the anaerobic chamber. Samples were processed and stained anaerobically, using reduced media.

**Figure 2.**
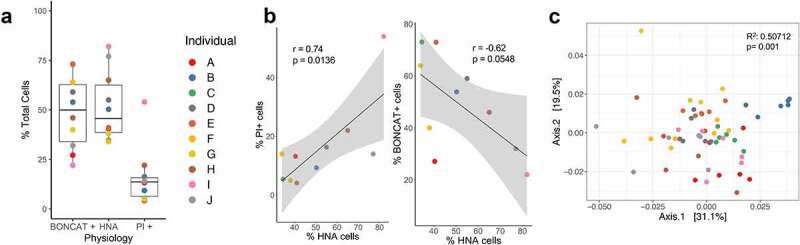
Abundance and diversity of physiological fractions per individual. (a) Relative abundance of cells in each physiological fraction (n = 10). b) Correlations between the proportion of PI+ bacteria and BONCAT+ bacteria to the proportion of HNA. C) PCoA of weighted UniFrac distances of regularized log transformed data indicate significant clustering by individual. There is significant clustering by (c) individual

**Figure 3.**
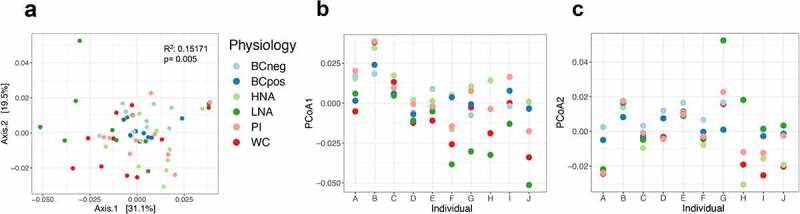
Beta diversity by physiological fractions. (a) PCoA of weighted UniFrac distances of regularized log transformed data indicate significant clustering by physiology. As there is large variation across individuals, the first (b) and second (c) principle components were plotted by individual to show trends in clustering by physiology

The proportion of each physiological fraction from total quantified cells was determined through flow cytometry. The HNA bacteria average 51.73 ± 17.59%, BONCAT+ at 49.01 ± 18.54%, and PI+ at 15.73 ± 14.58% (Figure 2a). There is a high correlation between the proportion of HNA and the proportion of PI+ bacteria (r = 0.74, *p* = .0136) and a borderline significant negative correlation between the proportion of HNA and the proportion of BONCAT+ bacteria (r = −0.62, *p* = .0548) (Figure 2b). 16S rRNA gene amplification and sequencing identified that these physiological cell fractions are distinct from one another and from the whole community. Principal coordinate analysis on pairwise weighted UniFrac distances shows that samples cluster strongly by individual stool donor (R^2^ = 0.50712, *p* = .001, PERMANOVA) (Figure 2c), and secondarily based on physiology (R^2^ = 0.15171, *p* = .005, PERMANOVA) (Figure 3a). When broken down by individual, HNA and LNA appear most distinct from one another on the first principal component, and BONCAT- most distinct from the other samples on the second principal component (Figure 3bc).

**Figure 4.**
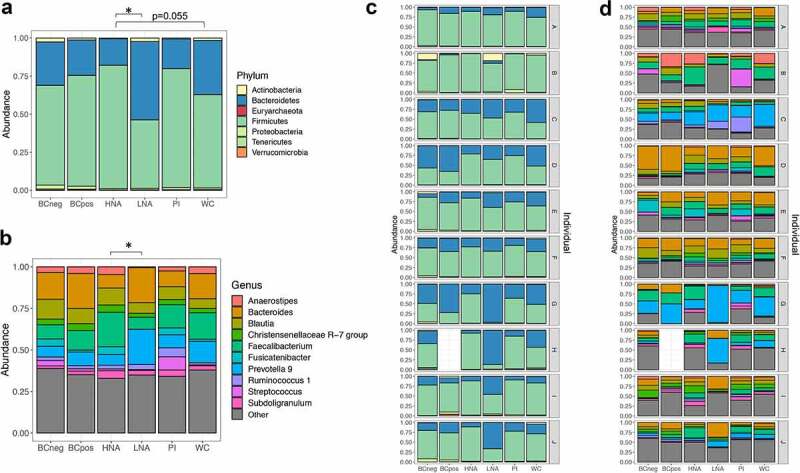
Taxonomic overview of the physiologically distinct subpopulations of the human gut microbiota. Relative abundance at the (a) phylum level and (b) top ten genera across physiological groups and by individual (c) and (d). Pairwise Adonis tests, with FDR adjustment

We next set out to determine specific differences between physiological fractions. The HNA and LNA communities are significantly different from one another at the phylum and genus level (*p* < .05, PERMANOVA with FDR adjustment) and the HNA is borderline significantly different from the whole community at the phylum level (*p* = .055, PERMANOVA with FDR adjustment) (Figure 4a and b). No other fractions were significantly different from one another after FDR adjustment. Phylum and genus level relative abundances are broken down by individual as well (Fig 4cd). To determine specifically which taxa are differentially abundant, we fitted a count regression for correlated observations with the beta-binomial model (Corncob) to every taxa and compared each physiological fraction to the whole community or to their low/high activity counterpart.^46^ The HNA fraction is dominated by the Firmicutes, averaging 80.98% of the HNA community compared to 61.28% of the whole community (*p* = .004, corncob). The HNA fraction also contains fewer Actinobacteria compared to the whole community (*p* = .0008, corncob). Similarly, the PI+ fraction resembles HNA more closely than it does the whole community, with significantly more Firmicutes at 78.02% (*p* = .04, corncob) and fewer Actinobacteria (*p* = .017, corncob). Conversely, the LNA fraction is more similar to the whole community, with 44.94% Firmicutes and 51.36% Bacteroidetes. As shown previously in the human gut,^10^ the HNA and LNA fractions contain the same bacteria (unweighted UniFrac *p* = .6), but present at different relative abundances (weighted UniFrac *p* = .028,), suggesting that nucleic acid content reflects bacterial physiology rather than taxonomy.

Surprisingly, the BONCAT+ and BONCAT- fractions are not significantly different from one another, or from the initial community (PERMANOVA). Still, Proteobacteria are significantly increased in the BONCAT- fraction compared to the whole community. While the BONCAT- fraction contains more Proteobacteria in general (*p* = .0069, corncob), the BONCAT+ fraction contain higher abundances of specific lineages within Proteobacteria such as *E. coli/Shigella* (*p* = .045, corncob), as well as differentially abundant *Coprococcus* 1 (*p* = 3.98e-05, corncob).

### The HNA bacteria of the gut microbiota contain more core taxa

**Figure 5.**
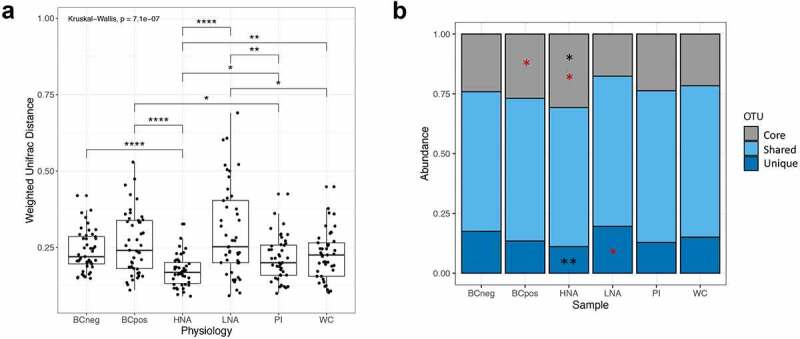
Similarity of physiologically distinct fractions across individuals. a) Weighted UniFrac distances for each pair of samples of rlog transformed data. b) Distribution of core, unique and shared taxa across physiological fractions. Red stars represent significantly different dispersion, black stars represent significantly differential abundance as per corncob models

HNA bacteria have previously been characterized as the more active bacteria in a community. Yet we found that the HNA bacteria are not necessarily the protein-producing bacteria, as HNA and BONCAT+ cell fractions are taxonomically different from one another (R^2^ =0.11721, *p* =.014, q =0.07 FDR). We hypothesized that HNA and BONCAT+ bacteria each represent different aspects of bacterial activity, but as active members of the community, both should contain more members of the core microbiome (*i.e*. taxa commonly present in most people). Changes in the core bacteria or functional groups have been linked to changes in host phenotype,^47–50^ suggesting the core microbiome is actively responsible for host phenotype, potentially through the metabolites they produce. Conversely, the less active fraction (BONCAT- and LNA) would contain the transient, environmental bacteria that are unique to the individual and perhaps less adapted to survive in the gut environment.^41–44^ Consistent with our hypothesis, the HNA fraction is more similar across individuals than the whole community (*p* < .01), or the LNA community (*p* < .0001). The LNA fraction is more different across individuals than the whole community (*p* < .05, Kruskal–Wallis test with Dunn’s test for multiple comparisons of weighted UniFrac distances) (Figure 5a). However, we found no equivalent differences in the BONCAT communities across individuals.

*p < .05, **p < .01, ***p < .001, and ****p < .0001.

To expand on the idea that the active fractions are more similar across individuals and thus contain more of the common core bacteria, we compared the distribution of core, unique, and shared taxa across the physiologically distinct fractions (Figure 5b). Core taxa are defined as those found in all sampled individuals, unique taxa are defined as those present in only one individual, and shared represent the remaining taxa. With this definition of 838 bacterial amplicon sequencing variants (ASVs), 12 (1.4%) were found in all individuals and 477 (57%) ASVs found in only one individual. The remaining 349 ASVs (41.5%) are shared across some proportion of individuals. The core taxa are differentially dispersed in HNA and BONCAT+, while also being significantly increased in HNA (*p* < .05). HNA also has fewer unique bacteria (*p* < .01, corncob), without a difference in dispersion. These patterns remain when the definition of core bacteria is loosened to ASVs found in 8/10 individuals, where 32 (3.8%) ASVs are considered as core. The enrichment of core taxa and lack of unique taxa in HNA, but not BONCAT, suggests the HNA may be providing more of the core metabolic activity in the gut.

### Physiological information is more sensitive than whole community profiling

**Figure 6.**
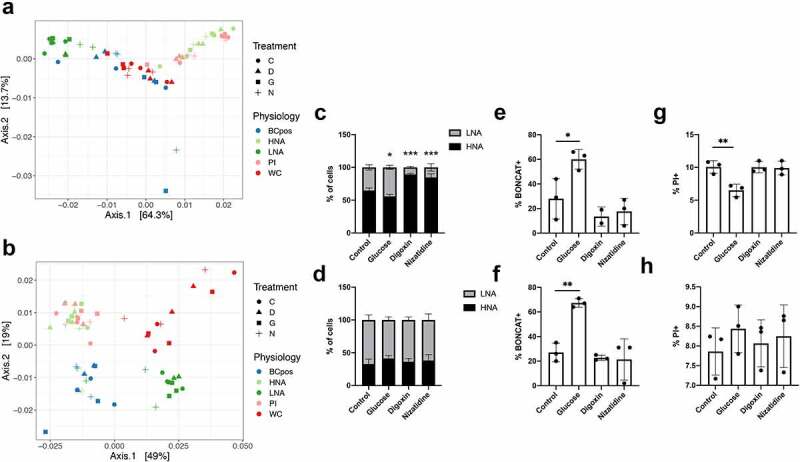
Differences of physiological groups after xenobiotic incubations. PCoA of rlog transformed weighted UniFrac distances broken down by physiology and treatment for a) individual 1 and b) individual 2. The circle for treatment C represent the control, the triangle for treatment D represents digoxin, the square for treatment G represents glucose, and the plus sign for treatment N represents nizatidine. Cellular relative abundance of (c) HNA and LNA, (e) BONCAT+, and (g) PI+ bacteria in Individual 1. The relative abundance of d) HNA and LNA, f) BONCAT+, and h) PI+ bacteria in Individual 2. Ex-vivo incubations were performed anaerobically at 37°C for 2 hours. N = 3 incubation replicates, error bars represent S.D, One-way ANOVA compared to the control group, with FDR correction

Focusing on the active subset of the gut microbiota allows for a finer resolution of changes in activity to be captured than whole-community DNA sequencing alone. To demonstrate this, we performed *in vitro* incubations of fecal samples from two individuals with various xenobiotics previously shown to alter the activity of the whole community (through metatranscriptomics), but not bacterial community composition (through 16S rRNA gene sequencing).^10^ We supplemented the BONCAT incubations with digoxin, nizatidine, or glucose, and compared the proportions of the physiological communities to a control incubation without any additions. PCoA plots of the weighted UniFrac distances, focusing on each of the two individuals separately, demonstrate a clear clustering by bacterial physiology (R^2^ = 0.65 and 0.64, respectively, Table 1) (Figure 6ab). Treatment alone has no effect; however, when looking at the effect of treatment nested within each physiological fraction, it has the largest effect size at R^2 ^= 0.75 (individual 1) and R^2 ^= 0.73 (individual 2) (Table 1). To determine specific pairwise effects of xenobiotic treatments relative to the controls in each physiological group in each individual, we compared Bray–Curtis dissimilarities. Specific changes in response to the xenobiotics in each physiological group were inconsistent, and limited by the low number of replicates per sample (n = 3). However, a few physiological groups trended toward significance compared to the control incubation. Specifically, in individual 1, the glucose incubation resulted in a change in beta diversity compared to control in the whole community and in the LNA fraction, and digoxin had effects in the PI+ fraction (*p* = .1, PERMANOVA). The taxonomic composition of these sorted fractions are in Figure S4.

**Table 1.** Effect size and dispersion effect of xenobiotics on the gut microbiota

	Individual 1				Individual 2			
	PERMANOVA		Dispersion		PERMANOVA		Dispersion	
	R^2^	p	F	p	R^2^	p	F	p
Physiology	0.65	0.001	6.25	0.001	0.64	0.001	1.3	0.275
Treatment	0.010	0.8	0.33	0.81	0.018	0.53	0.6635	0.582
Treatment %in% Physiology	0.75	0.001	0.6574	0.861	0.73	0.001	0.69	0.825

BONCAT-FACS-Seq provides actual cellular abundance data of each of the physiological fractions. The proportion of HNA bacteria decreased in individual 1 in response to glucose (65 ± 4% to 56 ± 3.2%), but increased in response to digoxin (to 89 ± 1.5%) and nizatidine (85 ± 5.5%) (Figure 6c); however, there were no changes in the proportion of HNA and LNA in individual 2 (Figure 6d). Increases in the proportion of HNA bacteria in individual 1 did not correspond to changes in composition, which remained similar to the PI+ fraction (Figure S4). The proportion of BONCAT+ bacteria increased in response to glucose in both individuals (28 ± 17% to 60 ± 7.9% and 27 ± 7.5% to 67 ± 3.5%, respectively) (Figure 6e and f). And lastly, the proportion of PI+ bacteria decreased in response to glucose in individual 1 (10 ± 0.96% to 6.5 ± 0.94%) but not individual 2 (Figures 6g and 6h). While the response in HNA/LNA and PI proportions is individual specific, there is a consistent increase in the proportion of BONCAT+ bacteria in response to glucose. Overall, we demonstrate how BONCAT-FACS with or without sequencing may be a faster and less expensive alternative to detect broad-level changes in bacterial activity than metatranscriptomics or whole-community profiling.

## Discussion

In this study, we have optimized the BONCAT-FACS-Seq protocol for the human gut microbiota to identify the translationally active bacteria. We then compared the cellular proportions and bacterial diversity of the BONCAT+ and BONCAT- fractions to the high and low nucleic acid containing bacteria (HNA and LNA), as well as the damaged fraction through PI staining. We found that the HNA and PI+ bacteria correlate well both in terms of abundance and diversity. The BONCAT+ and HNA fractions are taxonomically distinct, and as such we suggest they represent two different aspects of bacterial metabolic activity.

The high and low nucleic acid communities have been previously studied in the human gut microbiota. Previous work reported similar proportions of HNA, LNA, and PI in healthy individuals as our study, along with the dichotomous taxonomic distribution where the HNA are dominated by Firmicutes, and Bacteroidetes most abundant in the LNA fraction.^10^ This was found with different nucleic acid dyes, and both studies align with ours showing that the HNA fraction is taxonomically distinct from the whole community.^10,18^ Firmicutes have on average smaller genomes than the Bacteroidetes, suggesting genome size is not a factor in this bimodal distribution.^51,52^ In the same study, the Firmicutes were shown to be transcriptionally more active than the Bacteroidetes, and were the first to be damaged upon exposure to a perturbation,^10^ a characteristic of HNA that has been shown in other studies.^53,54^ The correlations seen with the relative abundance and diversity between HNA and PI suggest that a portion of the HNA bacteria are damaged, or contribute to the pool of damaged bacteria. However, the specific biological mechanisms at play that differentiate the HNA/LNA modality in the human gut remain undefined.

Relative nucleic acid content is thought to be related to the metabolic activity of the cell, as for example, it is supposed that a bacterial cell would contain more RNA when active than when inactive. SYBR Green I stains both DNA and RNA, and as such could follow multiple aspects of bacterial activity.^21^ As most RNA in the bacterial cell is involved in translation, we optimized a complementary technique, BONCAT to specifically probe the production of nascent proteins. BONCAT-FACS is a promising technique that allows for the sensitive, rapid identification of translationally active bacteria from just a short *in vitro* incubation. By adding excess HPG and incubating the bacteria in their own fecal slurry anaerobically, we maintained *in situ* conditions as much as possible. Thus, we were able to use a defined marker of activity: translation, to compare to an undefined marker of activity: relative nucleic acid content, to determine how much protein production contributes to the activity supposed by relative nucleic acid content.

Both relative nucleic acid content and BONCAT identified approximately half of the gut microbiota as active. This is similar to what has been seen in other studies using stable isotope probing with heavy water (D_2_O), where they found a range in the proportion of active bacteria from 30 to 76%.^16^ While the overall average proportion of HNA and BONCAT+ bacteria were similar in this study, their proportions did not correlate within an individual, and these two fractions are taxonomically distinct from one another. This suggests that the HNA bacteria are not necessarily the protein-producing bacteria within the system, and that HNA and BONCAT are two independent pools of bacteria. It is commonly assumed that transcription and translation are tightly coupled,^55^ so that both RNA and protein activities would be correlated and a relationship between BONCAT+ and HNA cells would exist. However, it has recently been shown that this tight coupling is not always the case, and specifically, there exists a substantial lag between transcription and translation across most Firmicutes.^56^ This is similar to what we are seeing in the human gut microbiome: there is no clear relationship between BONCAT+ and HNA cells, consistent with the notion that there is large variation in the relationship between transcription and translation across bacteria.

The HNA fraction is the physiological fraction most similar across individuals, containing more core taxa and less unique taxa than the whole community. Thus, the HNA cells may provide the bulk of the common cellular functions performed by the gut microbiota, containing the conserved functionality required for members of the gut microbiome to exist in the intestinal milieu. In this sense, the HNA fraction is metabolically active and still functionally relevant, but less resistant to damage than the BONCAT+ fraction.

The translationally active bacteria (BONCAT+) are not taxonomically distinct from the whole community or from the non-translating fraction (BONCAT-). Upon glucose addition, the proportion of BONCAT+ bacteria increased significantly, more than doubling in relative abundance. This suggests that most of the bacteria present in the gut have the potential to become translationally active, and that BONCAT with or without supplementation is able to differentiate between the actual and potential activity of the gut microbiota. Previous studies have suggested that approximately 20% of the gut microbiota is dormant, with 15% of the gut microbiome containing sporulation homologues.^45^ While the non-translating fraction determined in this study is larger than what has been predicted to be dormant, it may represent a bacterial reservoir; a spectrum ranging from dead, damaged, fully dormant, to a slower rate of protein production not captured with the incubation time used here. The lack of distinction between the BONCAT + and BONCAT- fractions could possibly be due to the leakiness in the sorting, but with purity levels ranging around 80%, only minor changes in the diversity between BONCAT+ and BONCAT- would be missed. As 16S rRNA gene sequencing rather than shotgun metagenomics was performed on the sorted fractions, differences might exist between the BONCAT + and BONCAT- fractions and the whole community at a higher taxonomic resolution.

Adding physiological information to sequencing data provides more sensitivity to subtle changes in the gut microbiota than whole-community diversity changes alone. The changes in the proportions of these physiological fractions in response to various drugs or glucose demonstrate the utility of a rapid method to determine changes in bacterial activity that occur before changes in taxonomic composition. The substantial increase in the proportion of BONCAT+ bacteria after glucose addition is in line with previous work,^15,16^ and demonstrates how the majority of the gut microbiota can be stimulated, differentiating between the realized potential of the community rather than the theoretical upper limit. BONCAT is a sensitive method to detect changes in the active fraction of the human gut microbiota, but the lack of changes in diversity of these fractions highlights the heterogeneity of activity in microbial communities seen elsewhere.^38^ Changes in the proportion of HNA bacteria in response to certain xenobiotics were not as consistent as changes in the BONCAT+ fraction, but could represent an increase in stress response based on the correlation with damaged bacteria.

We believe BONCAT is a suitable method to study the translationally active members of the human gut microbiota. The limitations of BONCAT are well described in this comprehensive review.^19^ Specific to this study, the combination of BONCAT with FACS-Seq remains an area to further optimize, as our sorting efficiency was lower than sorting SYBR-stained cells. It is possible that bacterial aggregates with a mixture of positive and negative cells are being sorted together as BONCAT+. Further optimization of the BONCAT-FACS protocol, for example to allow for double positive sorting could further enlighten the HNA/LNA distribution and its relationship to translational activity. Other measurements of activity, such as replication or transcription, are amenable to the gut microbiota incubation and click protocol, and would help further characterize the contribution of relative nucleic acid content as a physiologically marker of activity.

In conclusion, we compared two broad indicators of bacterial metabolism using cell sorting methods: relative nucleic acid content and translational activity. Both markers identify approximately half of the community as active, yet are distinct from one another. Thus, the HNA bacteria are not necessarily the protein-producing bacteria, and these two fractions represent distinct types of metabolism and activity. By focusing on the active subcommunities of the gut microbiota, we can more sensitively detect changes to perturbations than looking at the whole community alone. We hope this work lays the groundwork for using bulk-activity measurements to study how bacteria are able to change their physiology in response to various perturbations.

## Methods

### Sample collection

Human studies were performed with approval of the McGill Ethics Research Board (REB #A04-M27-15B). Ten healthy, unrelated individuals who had not taken antibiotics in the past 3 months and had not been diagnosed with a gastrointestinal condition provided fecal samples on site. Samples were immediately placed in the anaerobic chamber (Coy Laboratory Products, 5% H_2_, 20% CO_2_, 75% N_2_). Sample preparation and staining were performed in the anaerobic chamber; FACS was performed aerobically. Metadata questionnaires were completed post-sample donation, collecting information on dietary logs for the previous 48 hours, as well as recent travel history, antibiotic usage, and typical coffee, chocolate, tea, and dairy consumption.

### BONCAT incubations, growth curves, and click reaction

Gut microbiota sample preparation was prepared as previously describe.^57^ Bacteria were diluted 1/10 in 50% (v/v) of the supernatant retained from the first 6,000 x *g* centrifugation, 2 mM final concentration HPG, and remaining volume of rPBS. Bacteria were incubated at 37°C for 2 hours unless otherwise stated. When specified, glucose (0.2% final concentration) or drug additions (0.01 mg/ml final concentration) were added at the start of the BONCAT incubations. A no-HPG incubation is included as a control, and each sample is incubated in duplicate (triplicate for the xenobiotic experiments). Bacteria were fixed with 80% ethanol to a final concentration of 50% (v/v) and stored at 4°C until processed with the click reaction that same day.

For the click reaction, bacteria were pelleted and resuspended in the click reaction solution (Click-iT Cell buffer kit, ThermoFisher Scientific) containing 5 µM Alexa-647 azide, and incubated in the dark at room temperature for 30 minutes. A no-Alexa control is included. Samples were then centrifuged at 8,000 x *g* for 5 minutes, supernatant removed, and washed with 80% ethanol, and then centrifuged again and resuspended in PBS and stained with SYBR Green I. Growth curves were performed anaerobically in the dark in 96 well plates, with triplicates for each condition, using the BioTek Epoch 2 microplate spectrophotometer at OD_600_ with shaking before each measurement.

### Fluorescence-activated cell sorting (FACS) and cell counts

Cell sorting was performed on the FACSAria III (BD Bioscience) equipped with a 488 nm laser and the appropriate detection filters, using a 70 μm nozzle at 70 psi and at a flow rate that would lead to less than 5% coincidence events. Positively stained cells were determined from debris and unstained cells using unstained controls. A total of 180,000 events were sorted using a 70 μm nozzle for each population for each individual and frozen at −80 °C for later DNA extraction. Sheath fluid was collected at the end of every sorting day as a negative control to detect contaminant DNA. Data files were analyzed using FlowJo V7 software (FlowJo LLC). Cell count data was analyzed as previously reported.^57^

### DNA extraction and 16S gene amplicon bioinformatics analysis

Samples were stored at −80°C until DNA extraction. Samples were extracted with the Qiagen AllPrep PowerFecal kits as per the manufacturer’s instructions. The V4-V5 hypervariable region was amplified with the 515 F/926 R primers.^58^ Trimming, alignment of paired end reads, and quality filtering was performed by DADA2.^59^ Taxonomic alignment was performed with a pre-trained Naives Bayes classifier using SILVA 132 database on 99% OTUs using QIIME2.

Taxonomic and low abundance filtering was performed in phyloseq (v1.3) in R (v3.6.1). Reads present in the sheath fluid but absent in the whole community samples (69 ASVs) were removed. As well, ASVs without phyla-level taxonomic assignment were removed. A prevalence threshold was set at a minimum of 6 reads in at least 2 samples. Finally, two *Pseudomonas* ASVs were identified as contaminants and removed.

Count data was rlog transformed using the DESeq2 package (v1.26) and weighted UniFrac distance matrix was calculated using rbiom (v1.0). Beta diversity was assessed on weighted UniFrac distances using pairwise PERMANOVA with 999 permutations to test for significance using adonis in the vegan package (v2.5). Differential abundance testing was performed using the statistical analysis package corncob (v0.1) which performs beta-binomial regression models to determine differentially abundant and dispersed relative abundances. Weighted UniFrac distances between physiological groups were compared using the Kruskal–Wallis test in the rstatix package (v0.5). Alpha diversity of the sorted and unsorted comparisons was performed on samples rarified to 10,534 reads/sample without replacement using the Shannon diversity metric.

## Declarations

### Ethics approval and consent to participate

Informed consent was obtained from all volunteers. The study was approved by the McGill Ethics Research Board (REB #A04-M27-15B), Montreal, QC, Canada.

## Supplementary Material

Supplemental Material

## Data Availability

Bacterial 16S rRNA gene sequencing data can be accessed on the SRA database, accession number PRJNA661679. Code related to the analysis has been deposited in GitHub (https://github.com/MTaguer).

## References

[1] Petersen C, Round JL. Defining dysbiosis and its influence on host immunity and disease. Cell Microbiol. 2014;16(7):1024–15. doi:10.1111/cmi.12308. 24798552 PMC4143175

[2] Backhed F, Ley RE, Sonnenburg JL, Peterson DA, Gordon JI. Host-bacterial mutualism in the human intestine. Sci. 2005;307(5717):1915–1920. doi:10.1126/science.1104816. 15790844

[3] Duvallet C, Gibbons SM, Gurry T, Irizarry RA, Alm EJ. Meta-analysis of gut microbiome studies identifies disease-specific and shared responses. Nat Commun. 2017;8:1-10. 10.1038/s41467-017-01973-8 PMC5716994 29209090

[4] Gilbert JA, Quinn RA, Debelius J, Xu ZZ, Morton J, Garg N, Jansson JK, Dorrestein PC, Knight R. Microbiome-wide association studies link dynamic microbial consortia to disease. Nat. 2016;535:94–103. [Internet]. 10.1038/nature18850 27383984

[5] Mueller S, Saunier K, Hanisch C, Norin E, Alm L, Midtvedt T, Cresci A, Silvi S, Orpianesi C, Verdenelli M, et al. Differences in fecal microbiota in different european study populations in relation to age, gender, and country: a cross-sectional study. Appl Environ Microbiol. 2006;72(2):1027. doi:10.1128/AEM.72.2.1027-1033.2006. 16461645 PMC1392899

[6] Yatsunenko T, Rey FE, Manary MJ, Trehan I, Dominguez-Bello MG, Contreras M, Magris M, Hidalgo G, Baldassano RN, Anokhin AP, et al. Human gut microbiome viewed across age and geography. Nat. 2012;486(7402):222–227. doi:10.1038/nature11053. PMC3376388 22699611

[7] Schnorr SL, Candela M, Rampelli S, Centanni M, Consolandi C, Basaglia G, Turroni S, Biagi E, Peano C, Severgnini M, et al. Gut microbiome of the hadza hunter-gatherers. Nat Commun. 2014;5(1). doi:10.1038/ncomms4654. PMC3996546 24736369

[8] Falony G, Joossens M, Vieira-silva S, Wang J, Darzi Y, Faust K, Kurilshikov A, Bonder MJ, Valles-colomer M, Vandeputte D, et al. Population-level analysis of gut microbiome variation. Sci. 2016;352:560-564. 10.1126/science.aad3503 27126039

[9] Huttenhower C, Gevers D, Knight R, Abubucker S, Badger JH, Chinwalla AT, Creasy HH, Earl AM, FitzGerald MG, Fulton RS, et al. Structure, function and diversity of the healthy human microbiome. Nat. 2012;486:207–214. 10.1038/nature11234 PMC3564958 22699609

[10] Maurice CF, Haiser HJ, Turnbaugh PJ. Xenobiotics shape the physiology and gene expression of the active human gut microbiome. Cell. 2013;152(1–2):39–50. doi:10.1016/j.cell.2012.10.052. 23332745 PMC3552296

[11] Franzosa EA, Morgan XC, Segata N, Waldron L, Reyes J, Earl AM, Giannoukos G, Boylan MR, Ciulla D, Gevers D, et al. Relating the metatranscriptome and metagenome of the human gut. Proc Natl Acad Sci USA. 2014;111(22):E2329–38. doi:10.1073/pnas.1319284111. 24843156 PMC4050606

[12] Whidbey C, Sadler NC, Nair RN, Volk RF, Deleon AJ, Bramer LM, Fansler SJ, Hansen JR, Shukla AK, Jansson JK, et al. A probe-enabled approach for the selective isolation and characterization of functionally active subpopulations in the gut microbiome. J Am Chem Soc. 2019;141(1):42–47. doi:10.1021/jacs.8b09668. 30541282 PMC6533105

[13] Daniel H, Gholami AM, Berry D, Desmarchelier C, Hahne H, Loh G, Mondot S, Lepage P, Rothballer M, Walker A, et al. High-fat diet alters gut microbiota physiology in mice. Isme J. 2014;8(2):295–308. doi:10.1038/ismej.2013.155. 24030595 PMC3906816

[14] Heintz-Buschart A, Wilmes P. Human gut microbiome: function matters. Trends Ecol Evol. 2018;26:563–574. 10.1016/j.tim.2017.11.002 29173869

[15] Berry D, Mader E, Lee TK, Woebken D, Wang Y, Zhu D, Palatinszky M, Schintlmeister A, Schmid MC, Hanson BT, et al. Tracking heavy water (D2O) incorporation for identifying and sorting active microbial cells. Proc Natl Acad Sci. 2015;112(2):E194–203. [Internet]. doi:10.1073/pnas.1420406112. 25550518 PMC4299247

[16] Wang Y, Xu J, Kong L, Liu T, Yi L, Wang H, Huang WE, Zheng C. Raman–deuterium isotope probing to study metabolic activities of single bacterial cells in human intestinal microbiota. Microb Biotechnol. 2019;13(2):572–583. doi:10.1111/1751-7915.13519. 31821744 PMC7017835

[17] De Graaf AA, Maathuis A, De Waard P, Deutz NEP, Dijkema C, De Vos WM, Venema K. Profiling human gut bacterial metabolism and its kinetics using [U-13C]glucose and NMR. NMR Biomed. 2010;23(1):2–12. doi:10.1002/nbm.1418. 19593762

[18] Peris-Bondia F, Latorre A, Artacho A, Moya A, D’Auria G. the active human gut microbiota differs from the total microbiota. PLoS One. 2011;6:e22448. doi:10.1371/journal.pone.0022448. 21829462 PMC3145646

[19] Hatzenpichler R, Krukenberg V, Spietz RL, Jay ZJ. Next-generation physiology approaches to study microbiome function at single cell level. Nat Rev Microbiol. 2020;18(4):241–256. doi:10.1038/s41579-020-0323-1. 32055027 PMC7133793

[20] Gasol JM, Li Zweifel U, Peters F, Fuhrman JA, Hagström Å. Significance of size and nucleic acid content heterogeneity as measured by flow cytometry in natural planktonic bacteria. Appl Environ Microbiol. 1999;65(10):4475–4483. doi:10.1128/AEM.65.10.4475-4483.1999. 10508078 PMC91596

[21] Lebaron P, Parthuisot N, Catala P. Comparison of blue nuclei acid dyes for flow cytometric enumeration of bacteria in aquatic systems. Appl Environ Microbiol. 1998;64(5):1725–1730. doi:10.1128/AEM.64.5.1725-1730.1998. 9572943 PMC106222

[22] Lebaron P, Servais P, Agogué H, Courties C, Joux F. Does the high nucleic acid content of individual bacterial cells allow us to discriminate between active cells and inactive cells in aquatic systems? Appl Environ Microbiol. 2001;67(4):1775–1782. doi:10.1128/AEM.67.4.1775-1782.2001. 11282632 PMC92796

[23] Servais P, Casamayor EO, Courties C, Catala P, Parthuisot N, Lebaron P. Activity and diversity of bacterial cells with high and low nucleic acid content. Aquat Microb Ecol. 2003;33:41–51. doi:10.3354/ame033041.

[24] Morán XAG, Bode A, Suárez LÁ, Nogueira E. Assessing the relevance of nucleic acid content as an indicator of marine bacterial activity. Aquat Microb Ecol. 2007;46:141–152. doi:10.3354/ame046141.

[25] Bouvier T, Del Giorgio PA, Gasol JM. A comparative study of the cytometric characteristics of high and low nucleic-acid bacterioplankton cells from different aquatic ecosystems. Environ Microbiol. 2007;9(8):2050–2066. doi:10.1111/j.1462-2920.2007.01321.x. 17635549

[26] Bowman J, Amaral-Zettler L, Rich J, Luria C, Ducklow H. Bacterial community segmentation facilitates the prediction of ecosystem function along the western Antarctic Peninsula. Isme J. 2017;11(6):1460–1471. doi:10.1038/ismej.2016.204. 28106879 PMC5437343

[27] Wang Y, Hammes F, Boon N, Chami M, Egli T. Isolation and characterization of low nucleic acid (LNA)-content bacteria. Isme J. 2009;3(8):889–902. doi:10.1038/ismej.2009.46. 19421234

[28] Berney M, Vital M, Hu I, Weilenmann H, Egli T, Hammes F. Rapid, cultivation-independent assessment of microbial viability in drinking water. Water Res. 2008;42:4010–4018. doi:10.1016/j.watres.2008.07.017. 18694583

[29] Longnecker K, Sherr BF, Sherr EB. Activity and phylogenetic diversity of bacterial cells with high and low nucleic acid content and electron transport system activity in an upwelling ecosystem. Appl Environ Microbiol. 2005;71:7737–7749. doi:10.1128/AEM.71.12.7737-7749.2005. 16332746 PMC1317353

[30] Hatzenpichler R, Scheller S, Tavormina PL, Babin BM, Tirrell DA, Orphan VJ. In situ visualization of newly synthesized proteins in environmental microbes using amino acid tagging and click chemistry. Environ Microbiol. 2014;16(8):2568–2590. doi:10.1111/1462-2920.12436. 24571640 PMC4122687

[31] Samo TJ, Smriga SP, Malfatti F, Pedler BE, Azam F. Broad distribution and high proportion of protein synthesis active marine bacteria revealed by click chemistry at the single-cell level. Front Mar Sci. 2014;1:1–18.

[32] Hatzenpichler R, Connon SA, Goudeau D, Malmstrom RR, Woyke T, Orphan VJ. Visualizing in situ translational activity for identifying and sorting slow-growing archaeal−bacterial consortia. Proc Natl Acad Sci. 2016;113:E4069–78. 27357680 10.1073/pnas.1603757113 PMC4948357

[33] Sebastián M, Estrany M, Ruiz-González C, Forn I, Montserrat Sala M, Gasol JM, Marrasé C. High growth potential of long-term starved deep ocean opportunistic heterotrophic bacteria. Front Microbiol. 2019;10:1–12. doi:10.3389/fmicb.2019.00760. 31024513 PMC6468046

[34] Couradeau E, Sasse J, Goudeau D, Nath N, Hazen TC, Bowen BP, Chakraborty R, Malmstrom RR, Northen TR. Probing the active fraction of soil microbiomes using BONCAT-FACS. Nat Commun. 2019;10(1). doi:10.1038/s41467-019-10542-0. PMC6591230 31235780

[35] Valentini TD, Lucas SK, Binder KA, Cameron LC, Motl JA, Dunitz JM, Hunter RC. Bioorthogonal non-canonical amino acid tagging reveals translationally active subpopulations of the cystic fibrosis lung microbiota. Nat Commun. 2020;11(1):1–11. doi:10.1038/s41467-020-16163-2. 32385294 PMC7210995

[36] Geva-zatorsky N, Alvarez D, Hudak JE, Reading NC, Erturk-hasdemir D, Dasgupta S, Von AUH, Kasper DL. In vivo imaging and tracking of host – microbiota interactions via metabolic labeling of gut anaerobic bacteria. Nat Med. 2015;21:1091–1100. doi:10.1038/nm.3929. 26280120 PMC4694768

[37] Rigottier-Gois L, Bourhis A-G, Gramet G, Rochet V, Doré J. Fluorescent hybridisation combined with flow cytometry and hybridisation of total RNA to analyse the composition of microbial communities in human faeces using 16S rRNA probes. FEMS Microbiol Ecol. 2003;43:237–245. doi:10.1111/j.1574-6941.2003.tb01063.x. 19719684

[38] Reichart NJ, Jay ZJ, Hatzenpichler R, Krukenberg V, Parker AE, Spietz RL. Activity-based cell sorting reveals responses of uncultured archaea and bacteria to substrate amendment. Isme J. 2020;14(11):2851–2861. doi:10.1038/s41396-020-00749-1. 32887944 PMC7784905

[39] Lau JT, Whelan FJ, Herath I, Lee CH, Collins SM, Bercik P, Surette MG, Eckburg P, Bik E, Bernstein C, et al. Capturing the diversity of the human gut microbiota through culture-enriched molecular profiling. Genome Med. 2016;8(1):72. doi:10.1186/s13073-016-0327-7. 27363992 PMC4929786

[40] Kiick KL, Saxon E, Tirrell DA, Bertozzi CR. Incorporation of azides into recombinant proteins for chemoselective modification by the staudinger ligation. Proc Natl Acad Sci U S A. 2002;99:19–24. doi:10.1073/pnas.012583299. 11752401 PMC117506

[41] Zhang C, Derrien M, Levenez F, Brazeilles R, Ballal SA, Kim J, Degivry MC, Quéré G, Garault P, Van Hylckama Vlieg JET, et al. Ecological robustness of the gut microbiota in response to ingestion of transient food-borne microbes. Isme J. 2016;10:2235–2245. 26953599 10.1038/ismej.2016.13 PMC4989305

[42] Derrien M, Van Hylckama Vlieg JET. Fate, activity, and impact of ingested bacteria within the human gut microbiota. Trends Microbiol. 2015;23(6):354–366. doi:10.1016/j.tim.2015.03.002. 25840765

[43] David LA, Maurice CF, Carmody RN, Gootenberg DB, Button JE, Wolfe BE, Ling AV, Devlin AS, Varma Y, Fischbach MA, et al. Diet rapidly and reproducibly alters the human gut microbiome. Nat. 2014;505:559–563. doi:10.1038/nature12820. PMC3957428 24336217

[44] McNulty NP, Yatsunenko T, Hsiao A, Faith JJ, Muegge BD, Goodman AL, Henrissat B, Oozeer R, Cools-Portier S, Gobert G, et al. The impact of a consortium of fermented milk strains on the gut microbiome of gnotobiotic mice and monozygotic twins. Sci Transl Med. 2011;3(106):106ra106. doi:10.1126/scitranslmed.3002701. PMC3303609 22030749

[45] Lennon JT, Jones SE. Microbial seed banks: the ecological and evolutionary implications of dormancy. Nat Rev Microbiol. 2011;9:119–130. doi:10.1038/nrmicro2504. 21233850

[46] Martin BD, Witten D, Willis AD. Modeling microbial abundances and dysbiosis with beta-binomial regression. Ann Appl Stat. 2020;14:94–115. 32983313 10.1214/19-aoas1283 PMC7514055

[47] Salonen A, Salojärvi J, Lahti L, De Vos WM. The adult intestinal core microbiota is determined by analysis depth and health status. Clin Microbiol Infect. 2012;18:16–20. doi:10.1111/j.1469-0691.2012.03855.x. 22647042

[48] Chen RY, Kung VL, Das S, Hossain MS, Hibberd MC, Guruge J, Mahfuz M, Begum SMKN, Rahman MM, Fahim SM, et al. Linking the duodenal microbiota to stunting in a cohort of undernourished Bangladeshi children with enteropathy. N Engl J Med. 2020;383(4):321–333. doi:10.1056/NEJMoa1916004. 32706533 PMC7289524

[49] Turnbaugh PJ, Gordon JI. The core gut microbiome, energy balance and obesity. J Physiol. 2009;587(17):4153–4158. doi:10.1113/jphysiol.2009.174136. 19491241 PMC2754355

[50] Wang J, Lang T, Shen J, Dai J, Tian L, Wang X. Core gut bacteria analysis of healthy mice. Front Microbiol. 2019;10:1–14. doi:10.3389/fmicb.2019.00001. 31105675 PMC6491893

[51] Medvecky M, Cejkova D, Polansky O, Karasova D, Kubasova T, Cizek A, Rychlik I. Whole genome sequencing and function prediction of 133 gut anaerobes isolated from chicken caecum in pure cultures. BMC Genomics. 2018;19(1):561. doi:10.1186/s12864-018-4959-4. 30064352 PMC6069880

[52] Zou Y, Xue W, Luo G, Deng Z, Qin P, Guo R, Sun H, Xia Y, Liang S, Dai Y, et al. 1,520 reference genomes from cultivated human gut bacteria enable functional microbiome analyses. Nat Biotechnol. 2019;37(2):179–185. doi:10.1038/s41587-018-0008-8. 30718868 PMC6784896

[53] Ramseier MK, Von Gunten U, Freihofer P, Hammes F. Kinetics of membrane damage to high (HNA) and low (LNA) nucleic acid bacterial clusters in drinking water by ozone, chlorine, chlorine dioxide, monochloramine, ferrate(VI), and permanganate. Water Res. 2011;45(3):1490–1500. doi:10.1016/j.watres.2010.11.016. 21146846

[54] Lee Y, Imminger S, Czekalski N, Von Gunten U, Hammes F. Inactivation efficiency of Escherichia coli and autochthonous bacteria during ozonation of municipal wastewater effluents quantified with flow cytometry and adenosine tri-phosphate analyses. Water Res. 2016;101:617–627. doi:10.1016/j.watres.2016.05.089. 27322566

[55] Adhya S, Gottesman M. Control of transcription initiation. Annu Rev Biochem. 1978;47(1):967–996. doi:10.1146/annurev.bi.47.070178.004535. 354508

[56] Johnson GE, Lalanne JB, Peters ML, Li GW. Functionally uncoupled transcription–translation in bacillus subtilis. Nat. 2020;585(7823):124–128. doi:10.1038/s41586-020-2638-5. PMC7483943 32848247

[57] Maurice CF, Turnbaugh PJ. Quantifying and identifying the active and damaged subsets of indigenous microbial communities, 1st ed. Elsevier Inc. New York; 2013. 10.1016/B978-0-12-407863-5.00005-8 24060117

[58] Parada AE, Needham DM, Fuhrman JA. Every base matters: assessing small subunit rRNA primers for marine microbiomes with mock communities, time series and global field samples. Environ Microbiol. 2016;18(5):1403–1414. doi:10.1111/1462-2920.13023. 26271760

[59] Callahan BJ, Mcmurdie PJ, Rosen MJ, Han AW, Johnson AJA, Holmes SP. DADA2 : high-resolution sample inference from Illumina amplicon data. Nat Methods. 2016;13(7):581–583. doi:10.1038/nmeth.3869. 27214047 PMC4927377

