# Supplementary material for: Translational activity is uncoupled from nucleic acid content in bacterial cells of the human gut microbiota
Source: Gut Microbes. 2021 Mar 28;13(1):1903289. doi: 10.1080/19490976.2021.1903289 (PMC8009119; doi:10.1080/19490976.2021.1903289)
Supplement: Supplemental Material [file KGMI_A_1903289_SM6808.zip › Supplementary information/Supplementary Figures.docx]

**Supplementary Figure 1: Fluorescently activated cell sorting (FACS) of BONCAT samples.** Escherichia coli samples incubated **A)** with HPG but no Alexa and **B)** no HPG but with Alexa as gating controls, for **C)** E. coli incubated with HPG and clicked with Alexa. The gut microbiota **D)** incubated without HPG but with Alexa as a gating control for analysis with both HPG and Alexa in **E)**. Ethanol-fixed microbiota incubated with HPG and Alexa **F)** do not incorporate HPG compared to the same sample not fixed with ethanol **G)**. Q1 is SYBR Green negative and BONCAT positive, Q2 is SYBR Green positive and BONCAT positive. Q3 is SYBR Green positive and BONCAT negative. Q4 is SYBR Green negative and BONCAT negative.

**Supplementary Figure 2: Optimization of sorting to recapitulate the diversity of the initial population. A)** Anaerobic growth curves of sorted samples compared to an unsorted sample kept in anaerobic conditions. **B)** Between 50,000 and 1,000,000 bacterial events were sorted and 16S rRNA gene amplification by PCR of sorted and unsorted samples. **C)** Shannon’s diversity index of 16S rRNA gene sequencing of sorted and unsorted samples show a borderline significant decrease in alpha diversity (n=8, paired Wilcoxon rank test). **D)** Bray-Curtis PCoA of 16S rRNA gene sequencing results of sorted and unsorted samples for each individual.

**Supplementary Figure 3: Filtering based on negative control has minimal effect. A)** Phyla level read counts before any filtering was performed. **B)** Phyla level read counts after taxa present in the sheath fluid but absent from the unsorted sample (DNA) removed from all samples, as well as prevalence-based filtering. **C)** Genus level read count of the sheath fluid samples on the four sorting days. Note the differences in scale of abundance for panel C relative to panels A and B.

**Supplementary Figure 4: Relative abundances of phyla and family level taxa from 16S sequencing both individuals.** Sum averages from each of the n=3 incubation replicates for each xenobiotic.
