# Supplementary material for: Translational activity is uncoupled from nucleic acid content in bacterial cells of the human gut microbiota
Source: Gut Microbes. 2021 Mar 28;13(1):1903289. doi: 10.1080/19490976.2021.1903289 (PMC8009119; doi:10.1080/19490976.2021.1903289)
Supplement: Supplemental Material [file KGMI_A_1903289_SM6808.zip › Supplementary information/Table 1_Taguer_et_al.docx]

**Table 1: Methionine concentration in stool samples.** LC-MSMS concentrations of methionine in 5 fecal samples from unrelated healthy volunteers.

| Sample | Mean ± stdev uM/g |
| --- | --- |
| 1 | 113.3 ± 4.1 |
| 2 | 57.9 ± 0.1 |
| 3 | 71.2 ± 1.4 |
| 4 | 56.0 ± 1.4 |
| 5 | 128.0 ± 6.0 |
