# Supplementary material for: Translational activity is uncoupled from nucleic acid content in bacterial cells of the human gut microbiota
Source: Gut Microbes. 2021 Mar 28;13(1):1903289. doi: 10.1080/19490976.2021.1903289 (PMC8009119; doi:10.1080/19490976.2021.1903289)
Supplement: Supplemental Material [file KGMI_A_1903289_SM6808.zip › Supplementary information/Table 2_Taguer_et_al.docx]

**Table 2: Sorting purity**. Three BONCAT samples were acquired on the cell sorter, BONCAT+ and BONCAT- populations were sorted and individually reanalyzed on the sorter to determine sorting purity.

| Sample | Original | | Sorted BONCAT+ | | Sorted BONCAT- | |
| --- | --- | --- | --- | --- | --- | --- |
|  | BONCAT+ | BONCAT- | BONCAT+ | BONCAT- | BONCAT+ | BONCAT- |
| 1 | 49% | 51% | 71% | 29% | 1% | 99% |
| 2 | 64% | 36% | 90% | 10% | 16% | 84% |
| 3 | 30% | 70% | 79% | 21% | 0% | 100% |
| **SYBR Green I** | **Original** | | **Sorted HNA** | | **Sorted LNA** | |
|  | HNA | LNA | HNA | LNA | HNA | LNA |
|  | 64% | 36% | 93% | 7% | 2% | 98% |
