# Supplementary figures and images for: Translational activity is uncoupled from nucleic acid content in bacterial cells of the human gut microbiota
Source: Gut Microbes. 2021 Mar 28;13(1):1903289. doi: 10.1080/19490976.2021.1903289 (PMC8009119; doi:10.1080/19490976.2021.1903289)

# *Escherichia coli*

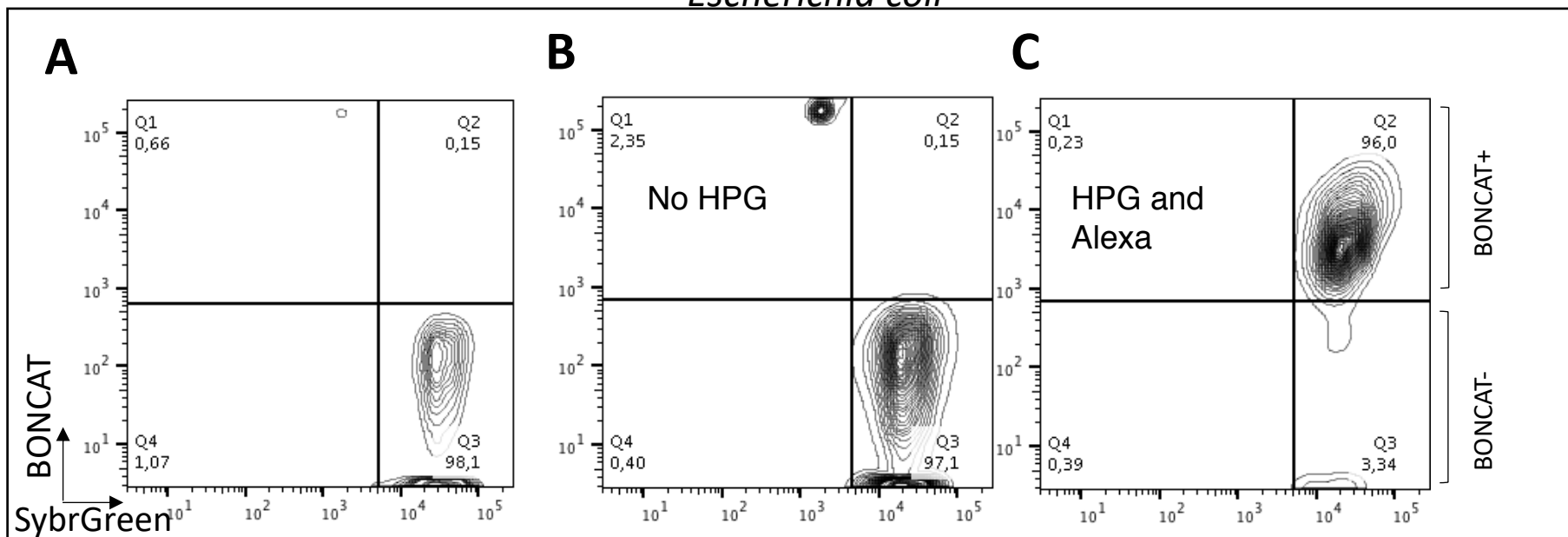

## Gut microbiota

## Dead

## Alive

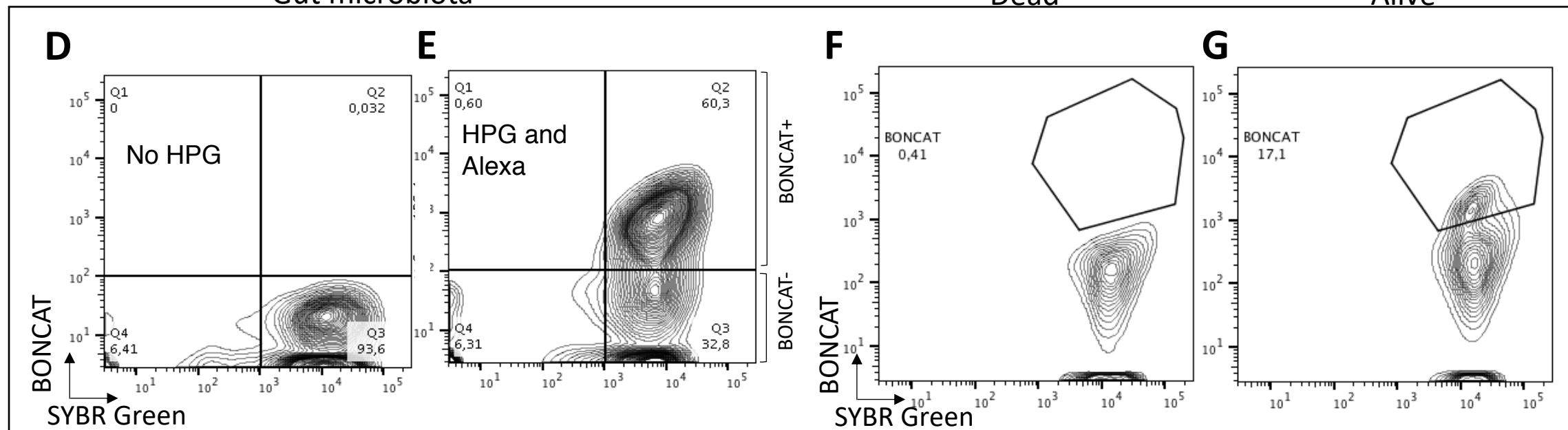

Supplement: Supplemental Material [file KGMI_A_1903289_SM6808.zip › Supplementary information/Figure S1_Taguer_et_al.pdf]

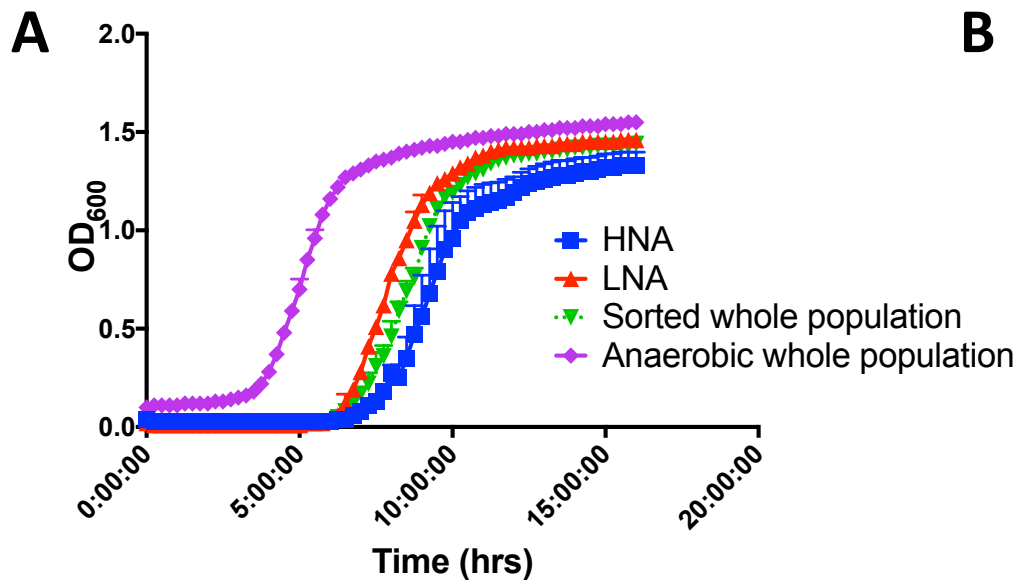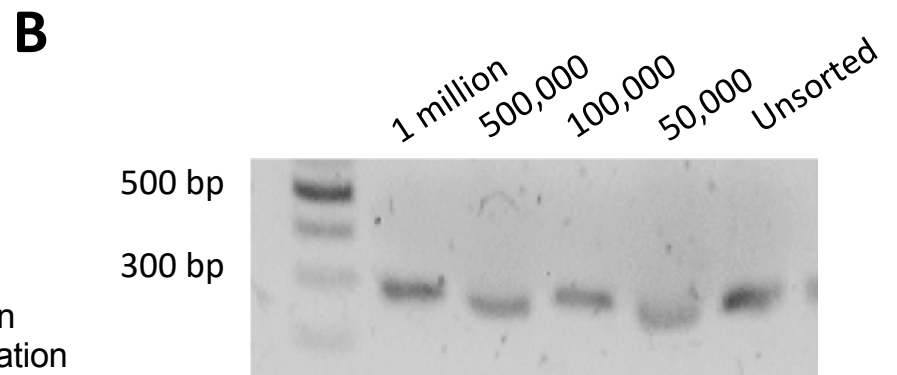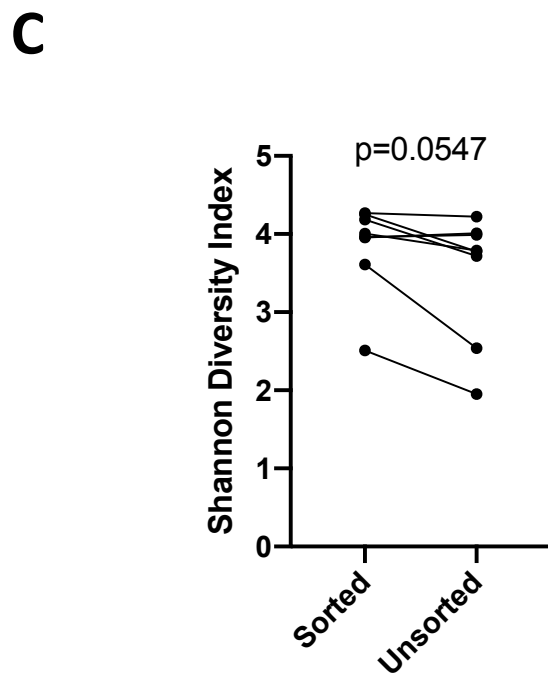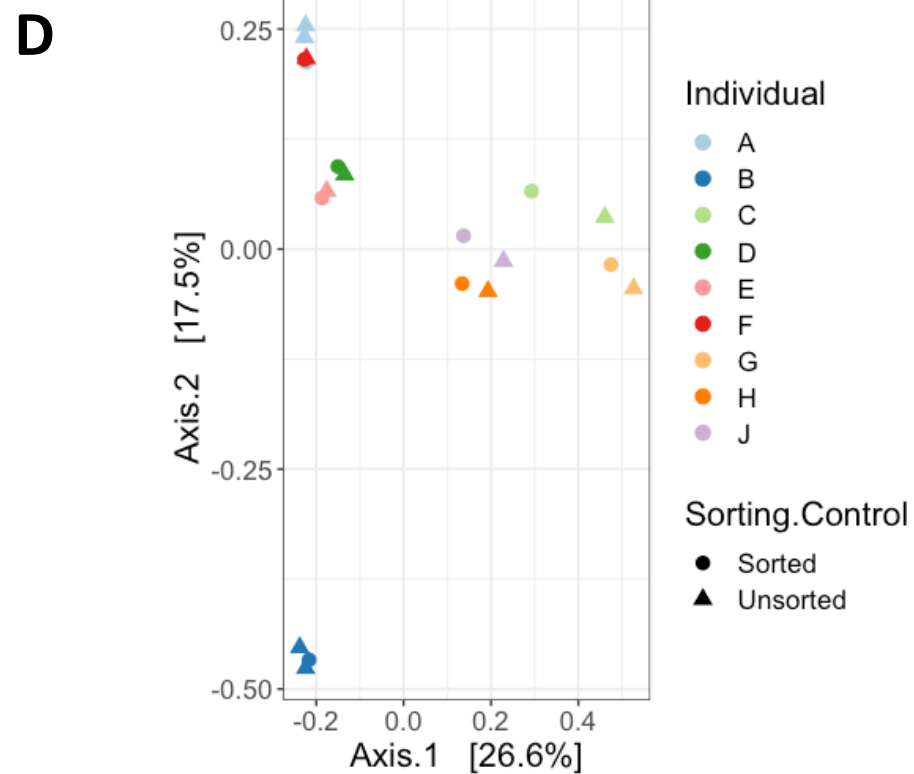

Supplement: Supplemental Material [file KGMI_A_1903289_SM6808.zip › Supplementary information/Figure S2_Taguer_et_al.pdf]

**A**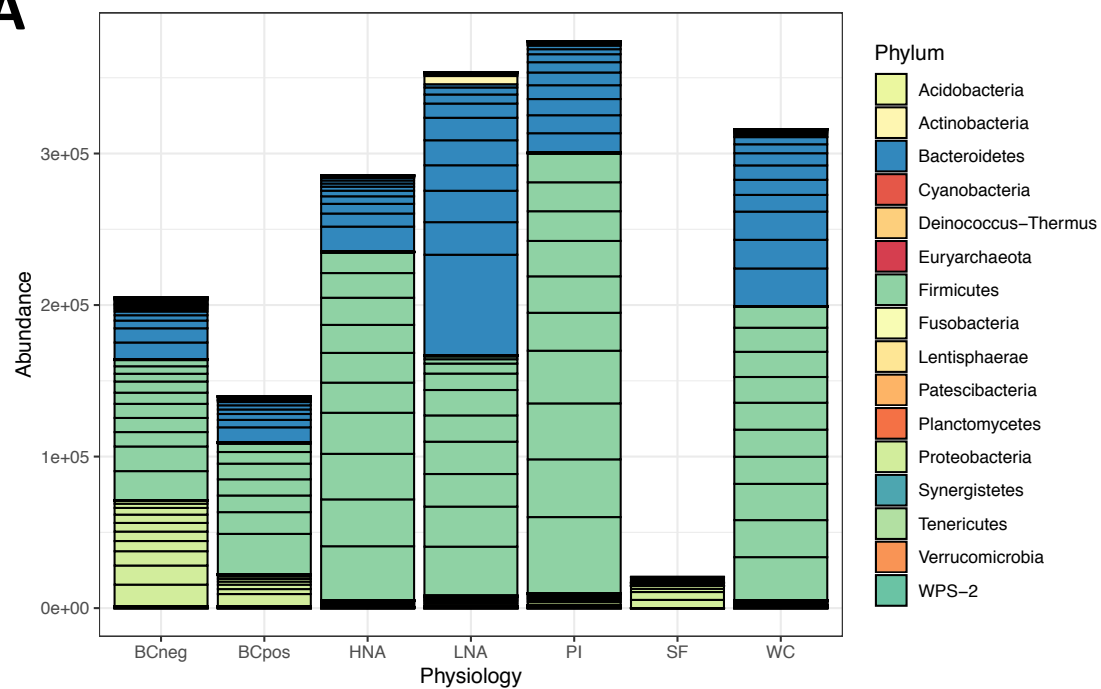**B**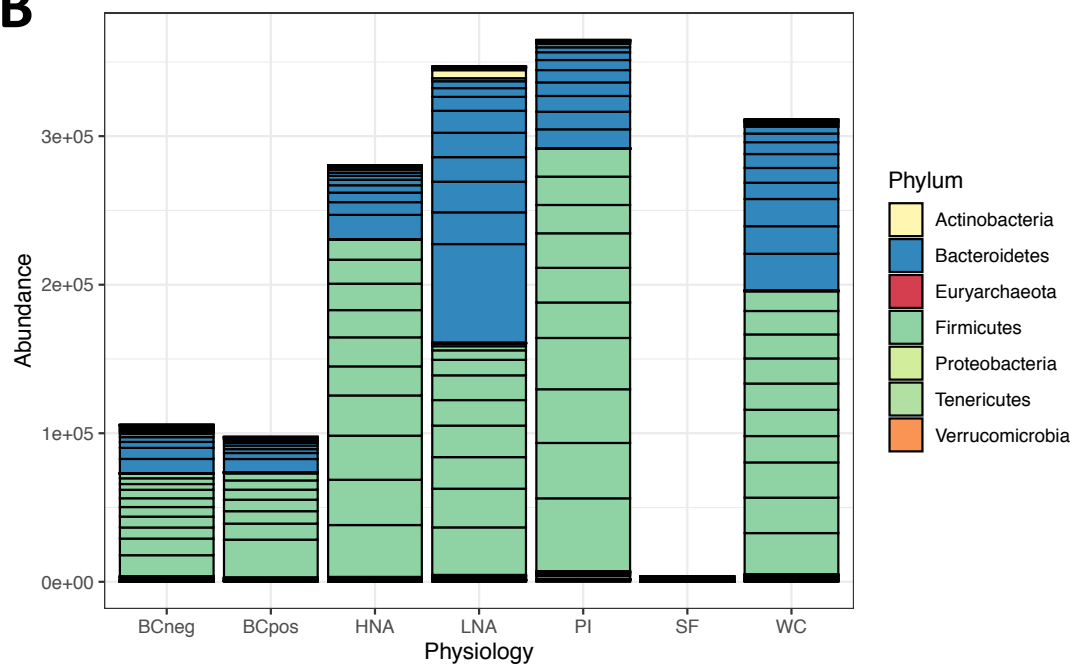**C**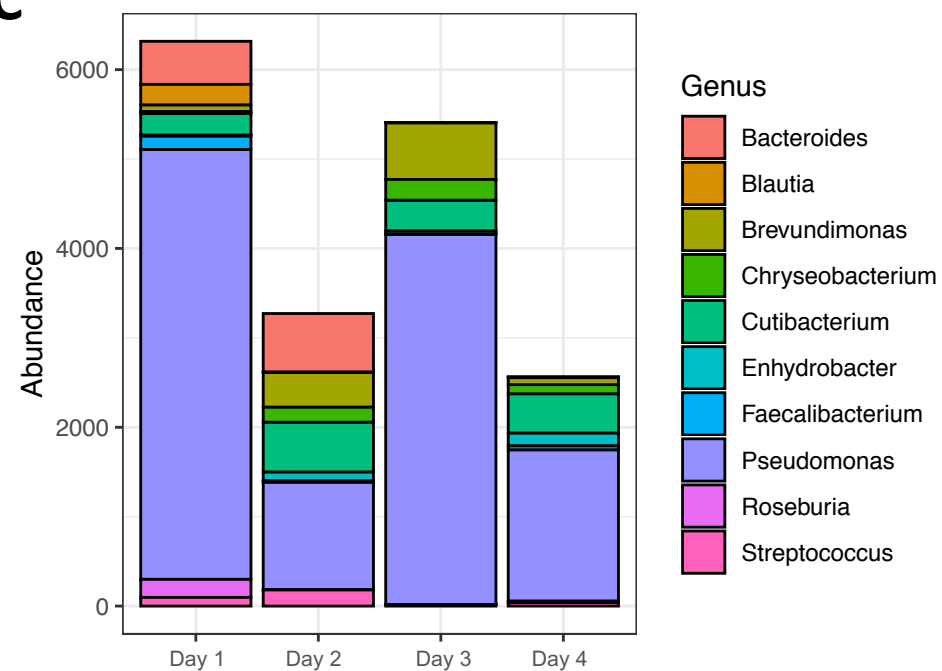

Supplement: Supplemental Material [file KGMI_A_1903289_SM6808.zip › Supplementary information/Figure S3_Taguer_et_al.pdf]

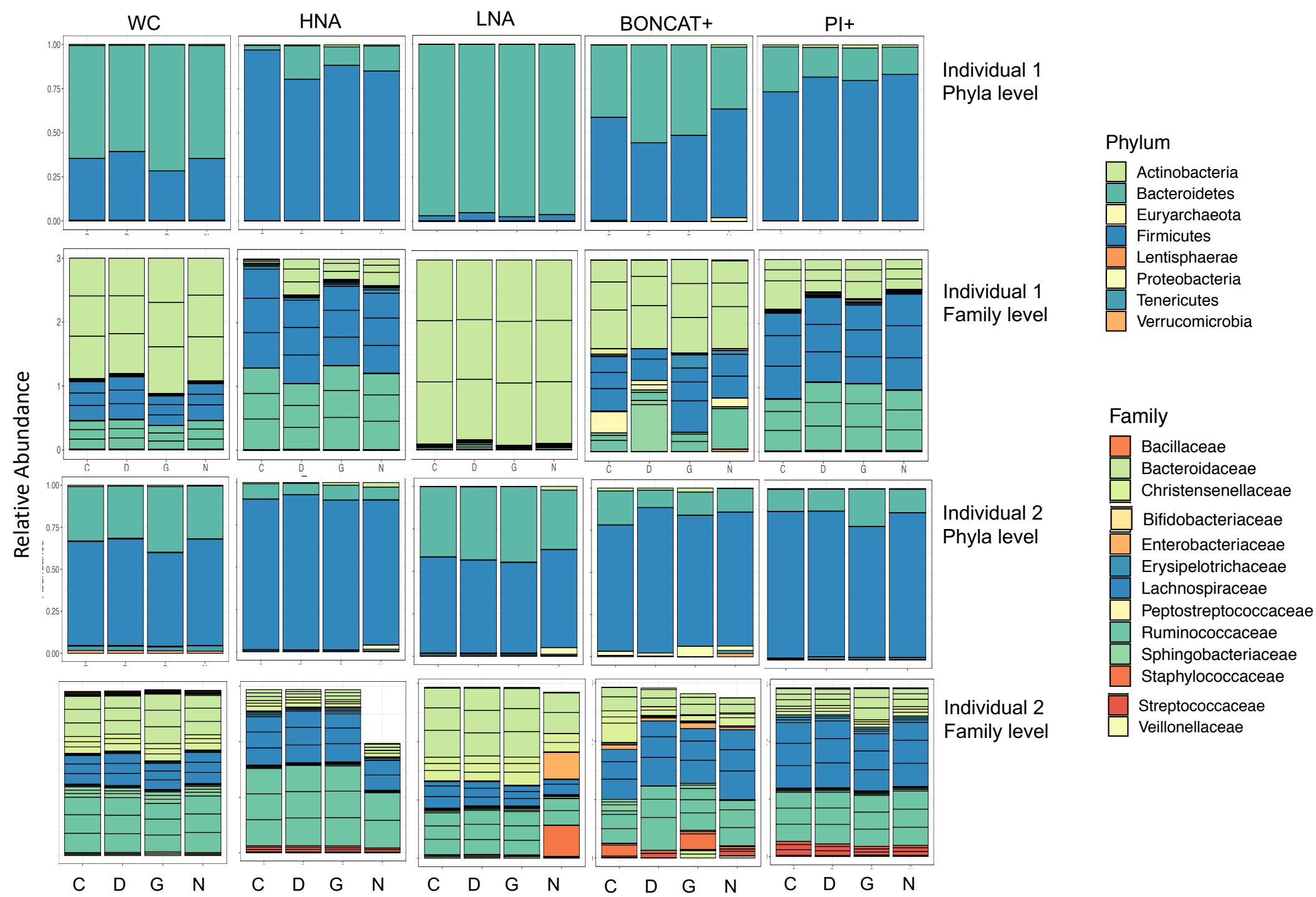

Supplement: Supplemental Material [file KGMI_A_1903289_SM6808.zip › Supplementary information/Figure S4_Taguer_et_al.pdf]
